# Morphological and biochemical variation of *Ajuga chamaecistus* Ging. ex Benth. in different habitats of Markazi province in the center of Iran

**DOI:** 10.1186/s12870-024-05125-1

**Published:** 2024-05-15

**Authors:** Fatemeh Mahmoodi, Mahdi Bikdeloo, Ali Khadivi, Morteza Akramian

**Affiliations:** 1 Department of Horticultural Sciences, Faculty of Agriculture and Natural Resources, Arak University, Arak, 38156-8-8349 Iran; 2 Department of Medicinal and Aromatic Plants, Faculty of Agriculture and Natural Resources, Arak University, Arak, 38156-8-8349 Iran

**Keywords:** *Ajuga chamaecistus*, Breeding, Morphology, Phenol, Antioxidant

## Abstract

**Background:**

Medicinal plants, such as *Ajuga chamaecistus* Ging. ex Benth. are a natural and available source of treatment for a wide range of diseases. The objective of the present study was to assess the morphological and biochemical variation of 70 accessions of this species collected from seven geographical areas of Markazi province in the center of Iran.

**Results:**

The measured traits exhibited considerable variability across the populations. Positive correlations were observed between antioxidant activity and total phenolic content, as well as total flavonoid content. Principal component analysis showed six components explaining 72.15% of the total variance, and the PC1 explained 20.68% of the total variance. The Ward dendrogram based on morphological variables identified two main clusters. Morphological analysis of *A. chamaecistus* showed a high variation between qualitative and quantitative traits that help the breeders for selecting the desired genotypes. The accessions collected from the Robat-Mil area showed the highest values for the recorded morphological characteristics. Also, the populations of Robat-Mil, Hassanabad, and Khaneh-Miran were characterized by high values of total phenolic content, total flavonoid content, and antioxidant activity, which can be used in various industries, including pharmaceuticals, cosmetics, and food.

**Conclusions:**

Overall, the present results showed that the best place for the growth of *A. chamaecistus* with the production of significant contents of phenol and flavonoid is in Robat-Mil area.

## Introduction

Medicinal plants are natural and available sources of treatment for a wide range of diseases, and since ancient times, these plants have played a significant role in the treatment of various diseases [1]. Nowadays, changing climatic conditions, uncontrolled grazing, and increasing demand for the consumption of medicinal plants pose a significant threat to their preservation in native habitats [2]. Approximately 8.00% of the world’s plant species (nearly more than 34,000 plants) are in a vulnerable and unstable state, which increases the risk of their erosion and destruction. Therefore, there is an urgent need for the protection and sustainable management of plant genetic resources [3]. Medicinal plants show high variations in their morphological and biochemical characteristics in different climates and natural habitats [4]. Therefore, for the effective use of medicinal plants, it is necessary to identify habitats and evaluate their morphological and biochemical characteristics.

The genus *Ajuga* L. belongs to the Ajugoideae subfamily of the Lamiaceae family, which is known for its rich secondary metabolites and diverse medicinal and aromatic properties. *Ajuga* species contain various bioactive compounds, including essential oils, flavonoids, triglycerides, phytosteroids, and diterpenes. These compounds often exhibit antioxidant activities and have been studied for their medicinal and pharmacological potential [5]. Many species of the *Ajuga* genus have ethnobotanical importance and are traditionally used to treat various ailments such as joint pain, sore throat, jaundice, fever, gout, asthma, gastrointestinal disorders, hemorrhoids, and diabetes [6]. Several studies have shown that *Ajuga* genus consists of 50 species with about 300 taxa, which are mostly distributed in the north temperate zone of the world [7]. In the flora of Iran, *Ajuga* is represented by 6 species and 8 infraspecific taxa [8]. One of them is *Ajuga chamaecistus* Ging. ex Benth. called Sefid-Moshkak in Farsi. It smells like musk and is a shrub and perennial herb that primarily grows on mountainous or rocky slopes at elevations ranging from 1850 to 2300 m. Its natural habitat is located in the western, central, and southern regions of Iran. It is also found in Afghanistan, eastern Turkey, the Caucasus, and Iraq [5].

*A. chamaecistus* has white stems and the height of the plant reaches 10 to 40 cm. The stem divisions from the base and the bush are much branched. Almost all the branches become hard and eventually become spiny. The leaf margin is dentate and covered with downy hairs. The calyx has a length between 5 and 10 mm, with different lengths of calyx teeth. The corolla is characterized by a combination of white and violet colors. The fruit is a nutlet, which can be seen in various forms of reticular wrinkled, with large holes. Its flowering season is generally in late spring [9, 10].

Due to the high medicinal value of *A. chamaecistus*, the natural population of this plant is currently facing significant threats from overexploitation by the local communities and destruction of habitats. A survey conducted on the species of Lamiaceae family in the flora of Iran has revealed the urgent need for conservation programs to protect this particular species [11]. To effectively conserve *A. chamaecistus*, a multidimensional approach is necessary. This approach should involve the selection of superior genotypes and the identification of optimal habitats. These efforts will contribute to the preservation and sustainable management of this valuable plant species.

There is no information regarding the morphological and biochemical variations of *A. chamaecistus* in Iran. Thus, the objective of this study was to investigate and characterize the morphological and biochemical variations among different populations of *A. chamaecistus* specifically grown in the Markazi province in the central region of Iran. Additionally, the study aimed to establish a connection between the two sets of data through the application of multiple statistical analyses. Also, the study aimed to explore the correlations between morphological and biochemical variation among populations which can help to identify accessions that can potentially serve as genetic resources for future breeding programs. By conducting this research, we can achieve a better understanding of the morphological and biochemical characteristics of *A. chamaecistus* in Iran and provide valuable insights for conservation programs.

## Materials and methods

### Plant material

**Fig. 1:**
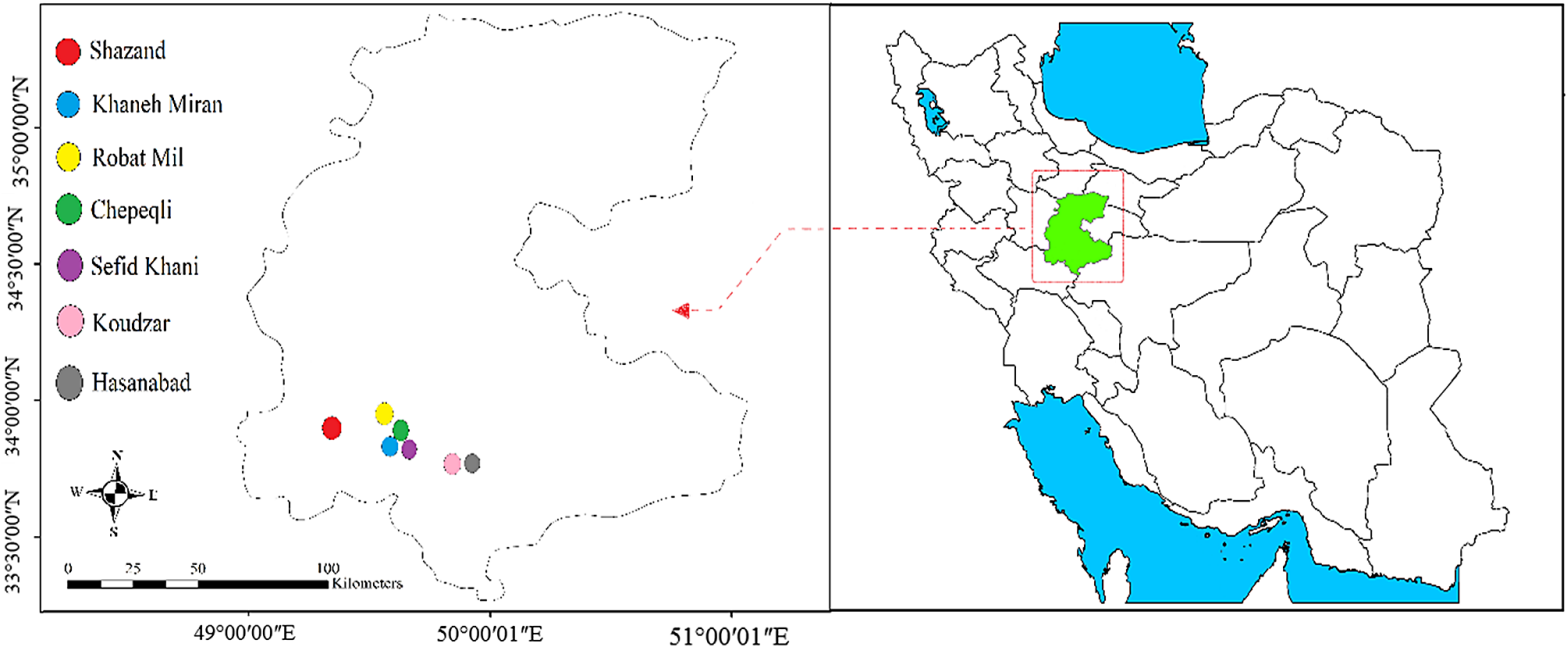
Geographical locations of the studied populations of *Ajuga chamaecistus* in Markazi province in the center of Iran

During June 2022, the aerial parts of 70 wild accessions of *A. chamaecistus* were collected from seven areas of Markazi province in Iran (Fig. 1) to study morphological and biochemical variation. The accessions were named based on their area (Fig. 1). Table 1 provides a summary of the geographical characteristics of the studied areas. The minimum distance between the accessions collected in each area was 200 m. The formal identification of the accessions was performed by Dr. Morteza Akramian based on Lamiaceae section of Flora of Iran [11]. Voucher accessions of this material have been deposited in the publicly available herbarium of Faculty of Agriculture and Natural Resources, Arak University, Iran with deposition number AUH-2348 to AUH-2354 (Table 1).

**Table 1:** Geographical distribution of *Ajuga chamaecistus* populations in Markazi province in the center of Iran

No.	Collection site	Longitude (E)	Latitude (*N*)	Altitude (m)	Sample size	Herbarium code
1	Shazand	49°30′36″	33°36′04″	2892	10	AUH-2348
2	Khaneh-Miran	49°57′17″	33°38′34″	2204	10	AUH-2349
3	Robat-Mil	49°53′06″	34°03′22″	2153	10	AUH-2350
4	Chepeqli	49°58′28″	33°46′18″	2545	10	AUH-2351
5	Sefid-Khani	49°58′29″	33°47′14″	2288	10	AUH-2352
6	Koudzar	49°43′34″	33°56′03″	2302	10	AUH-2353
7	Hasanabad	49°48′39″	33°57′50″	2062	10	AUH-2354

### Morphological characteristics

Twelve morphological traits were used to evaluate the morphological variation of the studied accessions (Table 2). Quantitative traits, such as leaf length, leaf width, internode length, and inflorescence length were measured using a ruler and a digital caliper. Also, qualitative traits were assessed using rating, scoring, and coding methods (Table 3). The Z-score method was used to standardize of both quantitative and qualitative characters.

**Table 2:** Descriptive statistics of quantitative morphological characteristics and chemical properties of the studied accessions *of Ajuga chamaecistus*

No.	Character	Unit	Min.	Max.	Mean	SD	CV (%)
1	Leaf length	cm	1.6	3.6	2.52	0.44	17.27
2	Leaf width	cm	0.3	0.9	0.54	0.17	31.53
3	Leaf length/leaf width	Ratio	2.37	8.66	4.99	1.44	28.77
4	Internode length	cm	0.9	2	1.46	0.26	17.93
5	Stem node number	Number	3	6	4.40	0.95	21.68
6	Inflorescence length	cm	1.8	3	2.32	0.23	9.80
7	Number of Inflorescences	Number	2	10	5.23	1.90	36.43
8	Total phenolic content	mg GAE/g DW	6.33	16.75	10.48	1.77	16.92
9	Total flavonoid content	mg QE/g DW	4.92	8.53	6.42	0.89	14.01
10	Antioxidant activity	mg ASA/g DW	2.13	7.52	4.22	1.34	31.79

**Table 3:** Frequency distribution for the measured qualitative morphological traits of *Ajuga chamaecistus*

		Frequency (no. of accessions)		
No	Trait	1	3	5
1	Flowering shoot color	Light green (20)	Green (39)	Dark green (11)
2	Leaf color	Light green (18)	Green (43)	Dark green (9)
3	Corolla color	Light purple (20)	Purple (38)	Dark purple (12)
4	State of stem hairs	Little (16)	Moderate (42)	High (12)
5	State of leaf hairs	Little (18)	Moderate (37)	High (15)

### Biochemical characteristics

#### Extraction method

All accessions collected from different geographical locations were air-dried at room temperature in the shade for one week. It was powdered and then passed through a 0.5 mm mesh screen to achieve a consistent particle size. Finally, 100 mg of dried powder of aerial parts was mixed with 10 ml methanol (80%) and extracted by shaking for 12 h. The obtained methanolic mixture was centrifuged at 8,000 rpm for 10 min. The supernatant was filtered through N. 1 Whatman filter paper and stored in a refrigerator at 4 °C until use.

#### Determination of total phenolic content

A modified Folin-Ciocalteu assay was used to determine the total phenolic content (TPC) of the samples [12]. This method is based on the reaction between phenolic compounds and the Folin-Ciocalteu reagent, which produces a blue color. The intensity of the color is directly proportional to the total phenolic content of the sample. Briefly, 400 µl of sample extracts was mixed with 2000 µl of Folin–Ciocalteu’s reagent (1:10) and 1600 µl of sodium carbonate (7.5%). The mixture was incubated for 30 min at room temperature in darkness. The absorbance of the resulting blue color was measured at 765 nm using a spectrophotometer. To determine the TPC, a calibration curve was prepared using gallic acid as a standard. The data were expressed as mg of gallic acid equivalents per g of dry matter (mg GAE/g DW).

#### Determination of total flavonoid content

The total flavonoid content (TFC) was estimated using the method of aluminium-chloride method [13]. In summary, 2 ml of the extracts were blended with 2 ml aluminum chloride (2%). The mixture was left at room temperature for 15 min and absorbance was measured at 415 nm. The results were performed according to the quercetin standard calibration curve and were expressed as mg quercetin equivalent per g of dry weight (mg QE g/ DW).

#### Assay of antioxidant activity

Antioxidant activity was determined using 1,1-diphenyl-2-picrylhydrazyl (DPPH) inhibitory activity with slight modifications [14]. Briefly, 1 ml of methanol extract of samples was added to 2 ml of DPPH methanol solution (0.1 mM). The mixture was shaken vigorously and kept at room temperature in the dark for 30 min. The absorbance of the samples was measured at 515 nm. The percentage of free radical inhibition activity of the extracts was calculated using the following equation.

$$\eqalign{ & \% {\rm{ }}\,DPPH\,{\rm{ }}radical\,{\rm{ }}inhibition\,{\rm{ }} = \cr & {\rm{ }}\,\left[ {\left( {{A_{control}} - {\rm{ }}{A_{sample}}} \right)/{A_{control}}} \right]{\rm{ }}\, \times {\rm{ }}\,100{\rm{ }} \cr}$$

Finally, the free radical inhibition ability of the samples was calculated using the standard curve prepared by ascorbic acid and the data were expressed as mg of ascorbic acid equivalents per g of dry matter (mg ASA/gDW).

### Statistical analysis

Experiments were conducted in triplicates. Descriptive statistics, including standard deviation, coefficients of variation (CV), mean, minimum, and maximum range of the characteristics were statistically analyzed using Excel 2019. Simple correlations between characteristics were determined utilizing Pearson correlation coefficients. Principal component analysis (PCA) was used to examine the relationship between accessions and determine the main characteristics using the SPSS software version 9. Cluster analysis was performed using Ward’s method and Euclidean distance coefficient using the PAST software version 4.

## Results and discussion

### Variation of traits

**Fig. 2:**
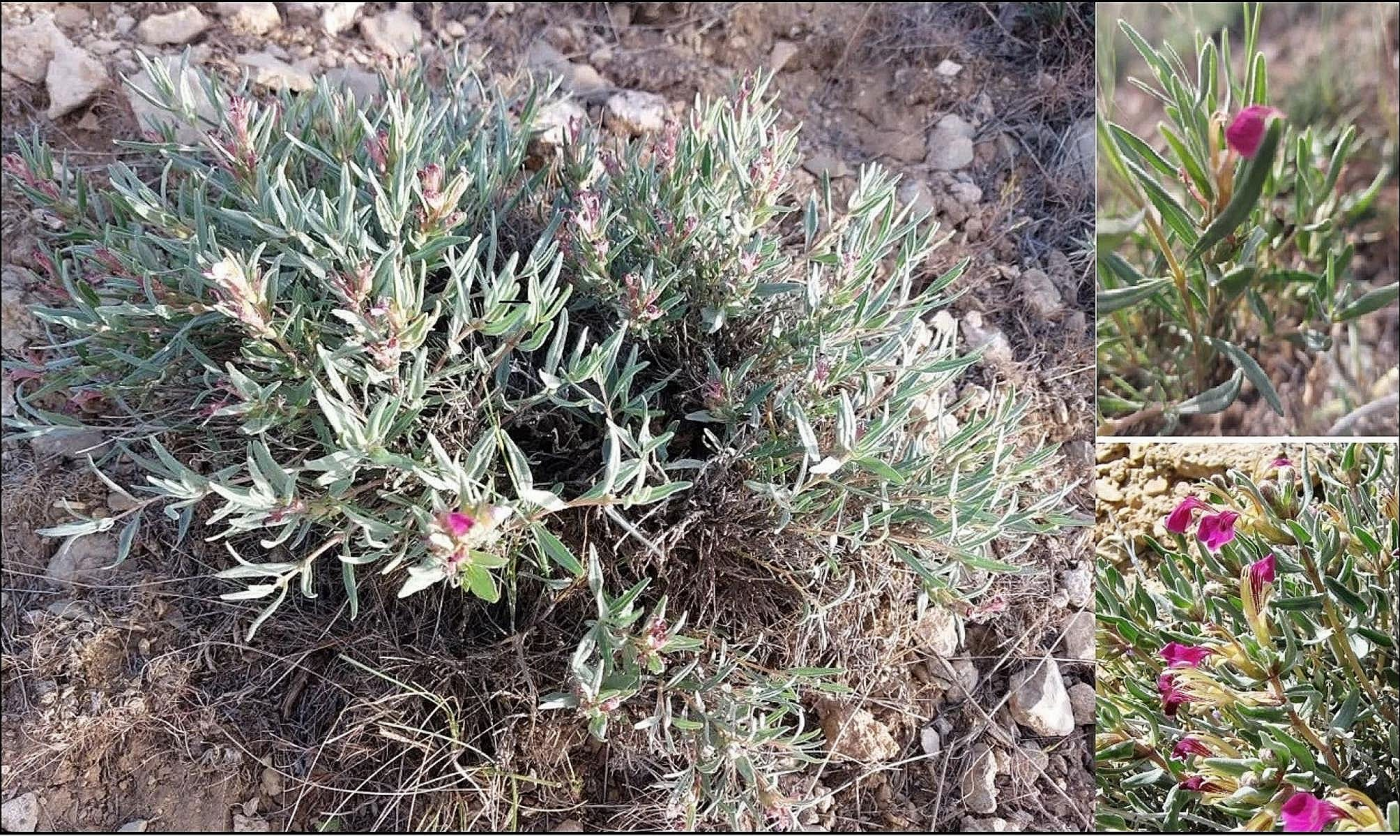
Aerial parts of *Ajuga chamaecistus* at the flowering stage in the natural habitat

The qualitative traits exhibited the highest coefficients of variation (CV), exceeding 40%. This indicates a significant amount of variation or differences in these traits among the populations. Additionally, several quantitative traits also showed relatively high CVs, with values of more than 20%. These traits include the number of inflorescences (36.43%), antioxidant activity (31.79%), leaf width (31.53%), the ratio of leaf length to width (28.77%), and stem node numbers (21.68%). The high CV suggest considerable variability in these traits across the populations. On the other hand, some traits showed lower CVs, indicating less variation among the populations. These traits include internode length (17.93%), leaf length (17.27%), total phenolic content (16.92%), total flavonoid content (14.01%), and inflorescence length (9.80%) (Table 2). These traits exhibited differences of less than 20% among the populations, suggesting a relatively lower level of variability. Overall, the CV provide a measure of the dispersion or variation within the accessions for each measured trait, with higher values indicating greater variability and lower values showing more homogeneity of traits among the accessions. These homogeneous traits can serve as reliable and consistent characteristics for identification or comparison purposes. On the other hand, when traits exhibit higher CV, exceeding 20%, it indicates a greater degree of variation between accessions, which can be useful in breeding programs or selection processes. Therefore, by considering the CV, researchers and breeders can identify stable and homogeneous traits as well as traits with higher variability, enabling them to make informed decisions regarding selection, breeding, and differentiation among accessions [15]. Leaf length ranged from 1.6 to 3.6 cm, and leaf width varied from 0.3 to 0.9 cm. Corolla color was light purple in 20, purple in 38, and dark purple in 12 accessions (Table 2). Leaf hairs content was predominantly moderate in the majority of accessions (37) (Table 3). Aerial parts of *A. chamaecistus* at the flowering stage in the natural habitat are showed in Fig. 2. Total phenolic content ranged from 6.33 to 16.75 mg GAE/g DW, total flavonoid content varied from 4.92 to 8.53 mg QE/g DW, and antioxidant activity ranged between 2.13 and 7.52 mg ASA/g DW (Table 2).

### Simple correlations between traits

**Fig. 3:**
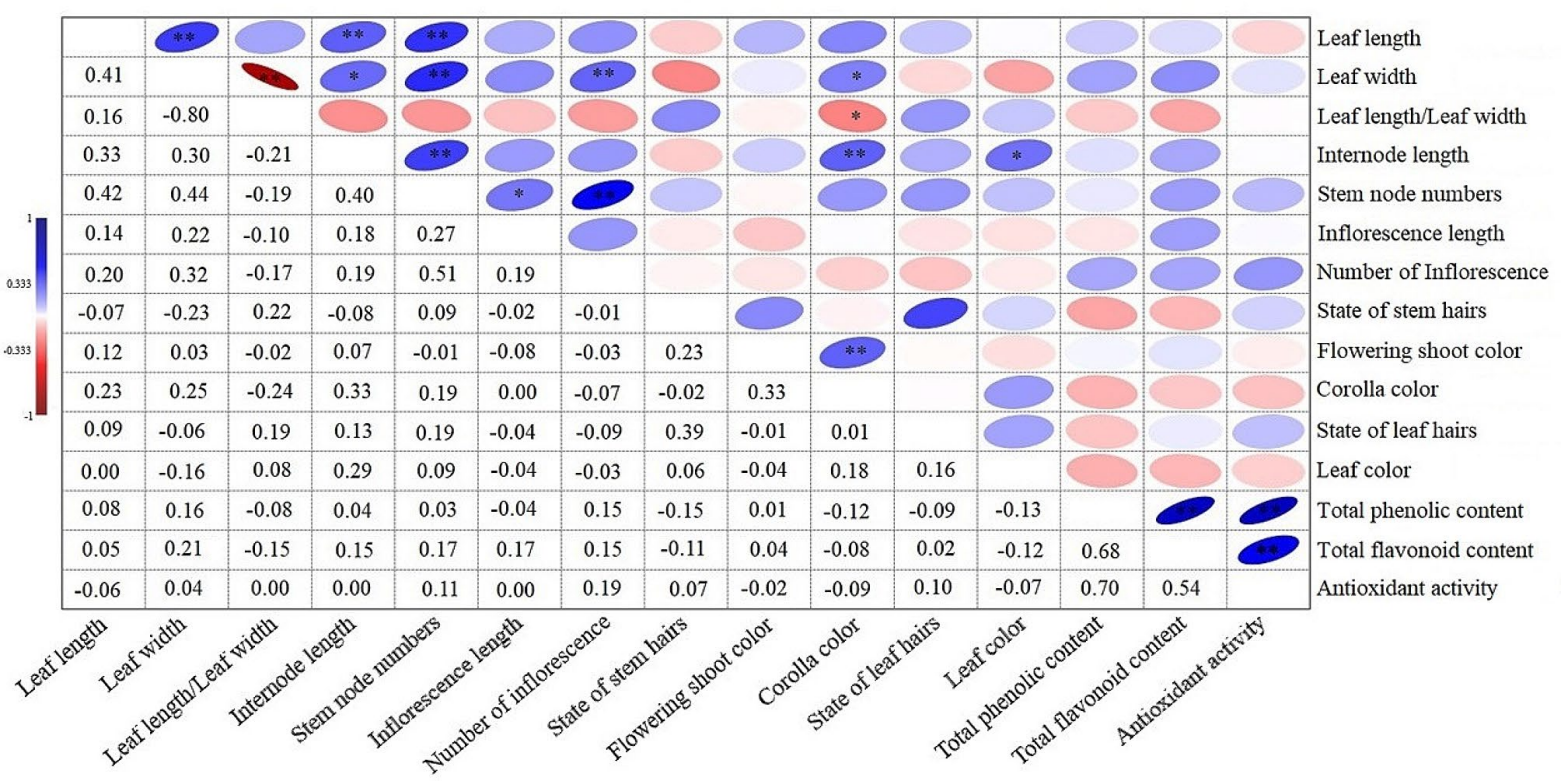
Correlation coefficients between the recorded characters in *Ajuga chamaecistus* populations

The obtained findings of the correlation performed using all morphological and biochemical characters are shown in Fig. 3. Leaf size (leaf length and width) showed positive correlations with internode length, stem node numbers, and the number of inflorescences. The presence of longer internodes and an increased number of nodes along the stem can contribute to overall plant height. Longer internodes provide structural support and allow leaves to be spaced out, reducing shading among foliage and maximizing light interception for photosynthesis. Therefore, it provides more resources to produce larger leaves and increase the number of inflorescences.

Also, the positive correlations were observed in the correlation matrix between antioxidant activity and total phenolic content, as well as total flavonoid content, is consistent with previous scientific reports [16, 17]. Phenolic compounds and flavonoids are well-known for their antioxidant properties, and one of their mechanisms of action is the ability to react with reactive oxygen species (ROS) such as lipid and hydroxyl radicals. Therefore, these compounds can scavenge harmful free radicals that can increase antioxidant activity [18].

### PCA

PCA was applied to identify the major sources of variation or relationships among the measured traits. By examining the principal components and their associated variance, we can identify which traits contribute the most to the observed patterns of variability. In the present study, PCA showed six components with explaining 72.15% of the total variance (Table 4). The PC1 explained 20.68% of the total observed variance and was correlated with leaf width, internode length, stem node numbers, and number of Inflorescences. These traits are associated with increasing the yield of vegetative biomass. The PC2 showed 15.17% of the total variance and was correlated with biochemical properties, including total phenolic content, total flavonoid content, and antioxidant activity. These traits are relevant to the chemical compounds in *A. chamaecistus* and these chemical compounds play a vital role in the medicinal properties of plants and have the potential to be utilized in various industries such as pharmaceuticals, cosmetics, and food. The PC3 showed 12.06% of the total variance and was correlated with leaf length/leaf width, state of stem hairs, and state of leaf hairs. In previous studies, the populations of different medicinal plant species such as *Satureja mutica* Fisch. & C. A. Mey, [19]. *Thymus daenensis* Celak, [20]. and *Zataria multiflora* Boiss [21]. were evaluated using PCA and their conclusions were similar to the present results.

**Table 4:** Principal component analysis of the studied characters in *Ajuga chamaecistus* accessions

Character	Component					
	1	2	3	4	5	6
Leaf length	0.47	0.33	0.24	-0.08	0.037	**0.64**
Leaf width	**0.80**	0.20	-0.35	0.05	0.17	-0.16
Leaf length/leaf width	-0.55	-0.11	**0.56**	-0.21	-0.08	0.53
Internode length	**0.56**	0.36	0.21	0.08	-0.39	-0.01
Stem node numbers	**0.68**	0.29	0.31	-0.29	0.11	-0.03
Inflorescence length	0.36	0.10	0.01	0.43	0.10	0.02
Number of Inflorescences	**0.56**	-0.01	0.07	-0.41	0.19	0.01
State of stem hairs	-0.21	0.16	**0.63**	0.06	0.48	-0.33
Flowering shoot color	0.06	0.17	0.18	**0.68**	0.41	0.22
Corolla color	0.28	0.51	0.01	**0.58**	-0.10	0.05
State of leaf hairs	-0.02	0.13	**0.68**	-0.04	0.06	-0.35
Leaf color	-0.04	0.33	0.34	0.05	**-0.67**	-0.22
Total phenolic content	0.45	**-0.75**	0.12	0.22	-0.14	0.12
Total flavonoid content	0.53	**-0.62**	0.17	0.13	-0.09	0.00
Antioxidant activity	-0.36	**0.70**	-0.34	0.14	0.05	0.11
Eigenvalue	3.10	2.28	1.81	1.39	1.16	1.09
% of Variance	20.68	15.17	12.06	9.23	7.77	7.24
Cumulative variance %	20.68	35.85	47.91	57.14	64.91	72.15

Bold values indicate the characteristic that most influence each PC

### Cluster analysis

**Fig. 4:**
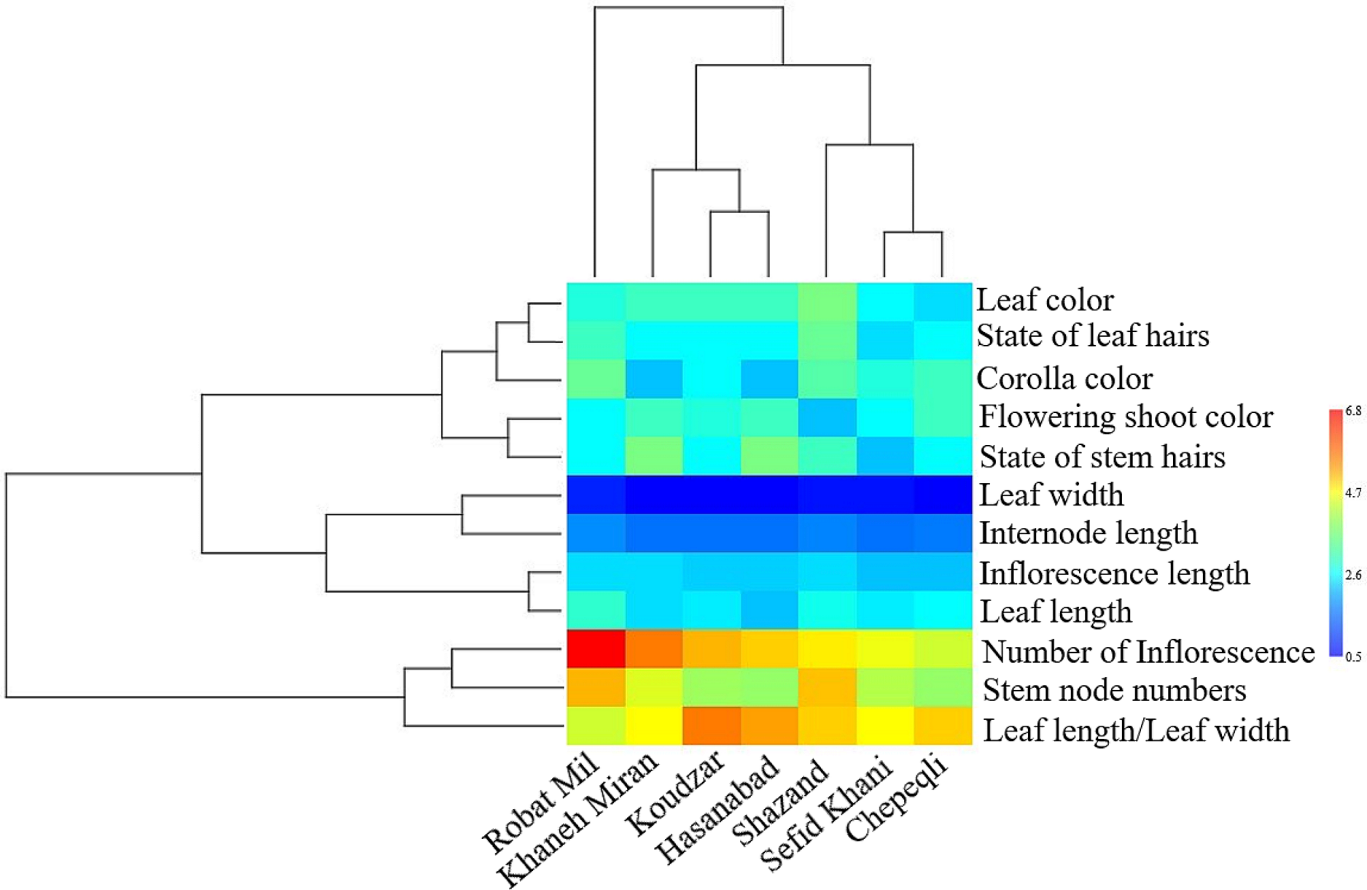
Cluster analysis for the studied populations of *Ajuga chamaecistus* using morphological traits. Mean values refer to colors from minimum displayed in dark blue to maximum represented in red

The Ward dendrogram based on morphological variables identified two main clusters (Fig. 4). The first cluster contained a population from Robat-Mil habitat, and this population was found to be superior in several traits, such as leaf length, leaf width, stem node numbers, internode length, number of inflorescences, and state of leaf hairs. These traits have a direct impact on enhancing yields in vegetative biomass. Additionally, the highest content of essential oil in *A. chamaecistus* was found to be produced and stored in the epidermal glands of the leaves and inflorescences. Therefore, populations that exhibit superiority in these traits can be utilized in breeding programs. The research also concluded that the climatic conditions of the Robat-Mil habitat in Iran are highly favorable for the growth of *A. chamaecistus*. This suggests that the species thrives and performs well in the specific environmental conditions found in that region. The second cluster was divided into two sub-clusters and the populations of each group were almost geographically close to each other. The first sub-cluster consisted of three populations from Khaneh-Miran, Koudzar, and Hasanabad. These populations exhibited similar values in leaf color, state of leaf hairs, inflorescence length, and internode length. The second sub-cluster contained three populations from the Shazand, Sefid-Khani, and Chepeqli habitats, which were similar in some traits including leaf length and leaf width. The obtained results are consistent with previous studies that have shown the influence of geographical conditions and ecological factors on the grouping of populations [22].

In a study on *Ajuga bracteosa* Wall ex Benth., plants grown at lower altitudes were found to be relatively taller and produced a greater number of leaves, as well as larger leaf dimensions and inflorescence numbers [23]. This suggests that *Ajuga bracteosa* individuals from lower altitudes tend to allocate more resources towards vegetative growth and reproduction. On the other hand, investigation on the morphological variability and phenolic content of *Ajuga iva* (L.) Schreb. collected from different geographical locations in Morocco showed that accessions collected from altitudes above 1000 m exhibited the highest values for various morphological characters and recorded the maximum total phenolic content [24]. This indicates that *Ajuga iva* individuals from higher altitudes may have adapted to harsher environmental conditions by producing more phenolic compounds, which can serve as protective antioxidants. These findings highlight the importance of considering ecological conditions, such as altitude and geographical location, when studying the growth and biochemical reactions of different *Ajuga* species. Environmental factors can have a significant impact on plant physiology and biochemistry, leading to variations in growth patterns and the production of secondary metabolites like phenolic compounds.

**Fig. 5:**
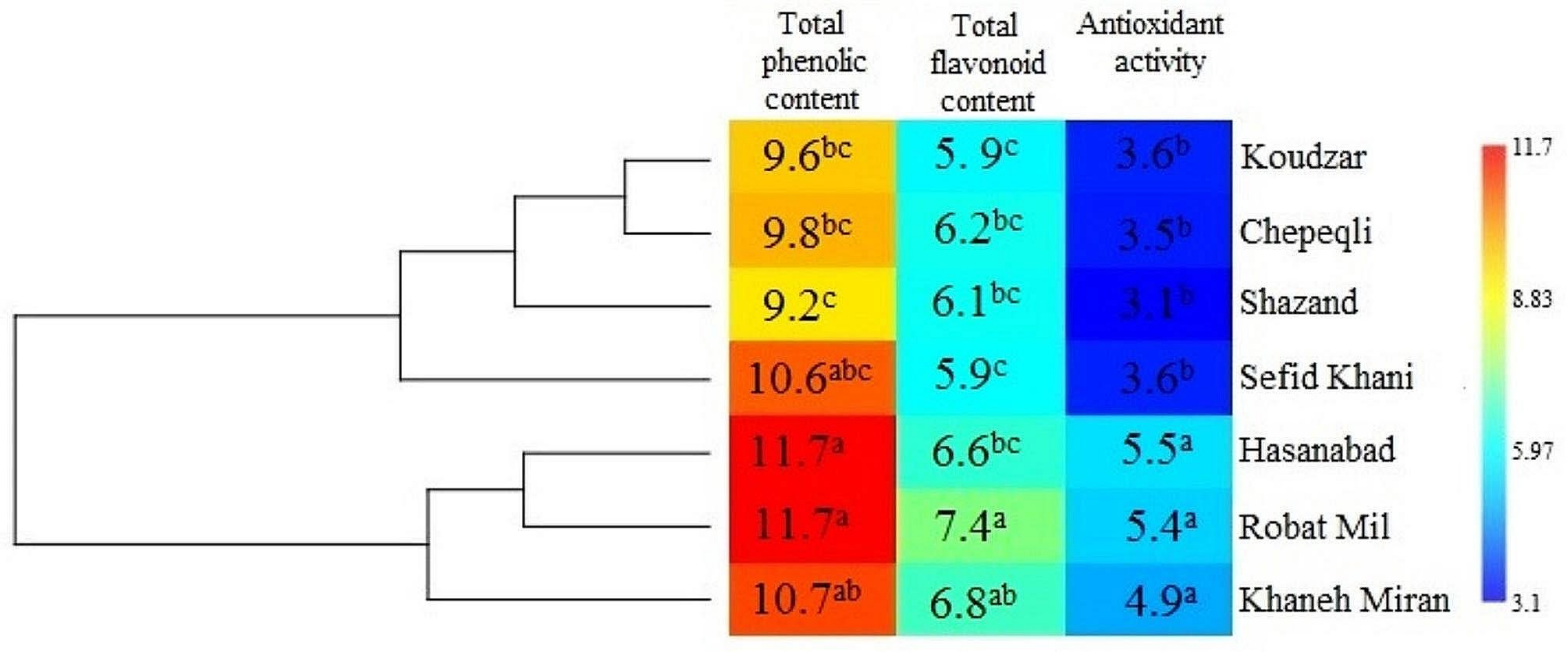
Cluster analysis for the studied populations of *Ajuga chamaecistus* using biochemical traits. Mean values refer to colors from minimum displayed in dark blue to maximum represented in red

**Fig. 6:**
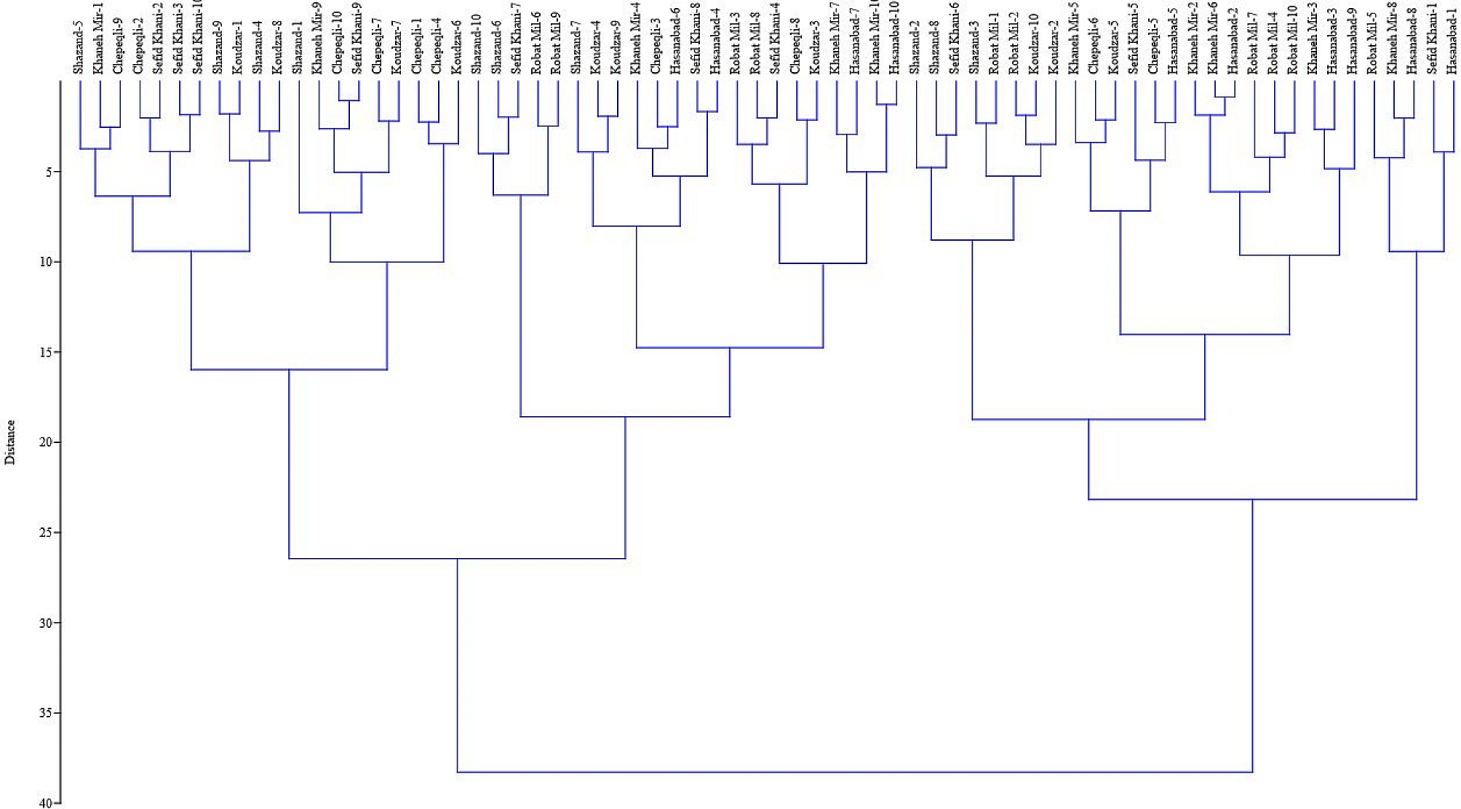
Cluster analysis for the studied accessions of *Ajuga chamaecistus* using Ward’s method based on morphological and biochemical traits

Also, the cluster analysis of biochemical properties showed two separate clusters (Fig. 5). The first cluster included four populations from Koudzar, Chepeqli, Shazand, and Sefid-Khani, which have relatively low amounts of total phenolic content, total flavonoid content, and antioxidant activity compared with the populations of the second cluster, which includes Hassanabad, Robat-Mil and Khaneh-Miran populations. Higher levels of total phenolic content and total flavonoid content indicate the potential presence of bioactive compounds with beneficial properties. Phenolic compounds and flavonoids are known for their antioxidant, anti-inflammatory, and antimicrobial activities, which are highly sought after in various industries. The antioxidant effect, free radical scavenging activity, and total phenolic content of aqueous and methanolic extracts of aerial parts of *A. chamaecistus* were investigated using ferric reducing antioxidant power, DPPH (2,2-diphenyl-1-picrylhydrazyl) test, and Folin-Ciocalteu methods [25]. The results showed that the butanol reaction had the highest phenolic content (26.5 mg GAE/g of extract) and the highest antioxidant power (346.7 mmol Fe/g of extract) and inhibited DPPH free radicals (IC50 = 15.34 µg/mL). Cluster analysis for the studied of *A. chamaecistus* using Ward’s method showed two major clusters with several sub-clusters (Fig. 6) based on morphological and biochemical traits, indicating high variation among the accessions.

Understanding the morphological and biochemical variation within *A. chamaecistus* populations has direct implications for potential breeding programs aimed at enhancing desirable traits. The identification of populations with unique biochemical profiles can guide the selection of individuals with high secondary metabolite production, which is often associated with medicinal properties. By selectively breeding individuals with superior biochemical profiles, it becomes possible to develop improved varieties with enhanced medicinal properties and increased yields. Furthermore, understanding the morphological variation can aid in the selection of individuals with desirable growth habits, disease resistance, and other important traits for cultivation purposes. These breeding efforts contribute to meeting the growing demand for medicinal plants and provide sustainable alternatives to wild harvesting [26].

### Biplot plot analysis

**Fig. 7:**
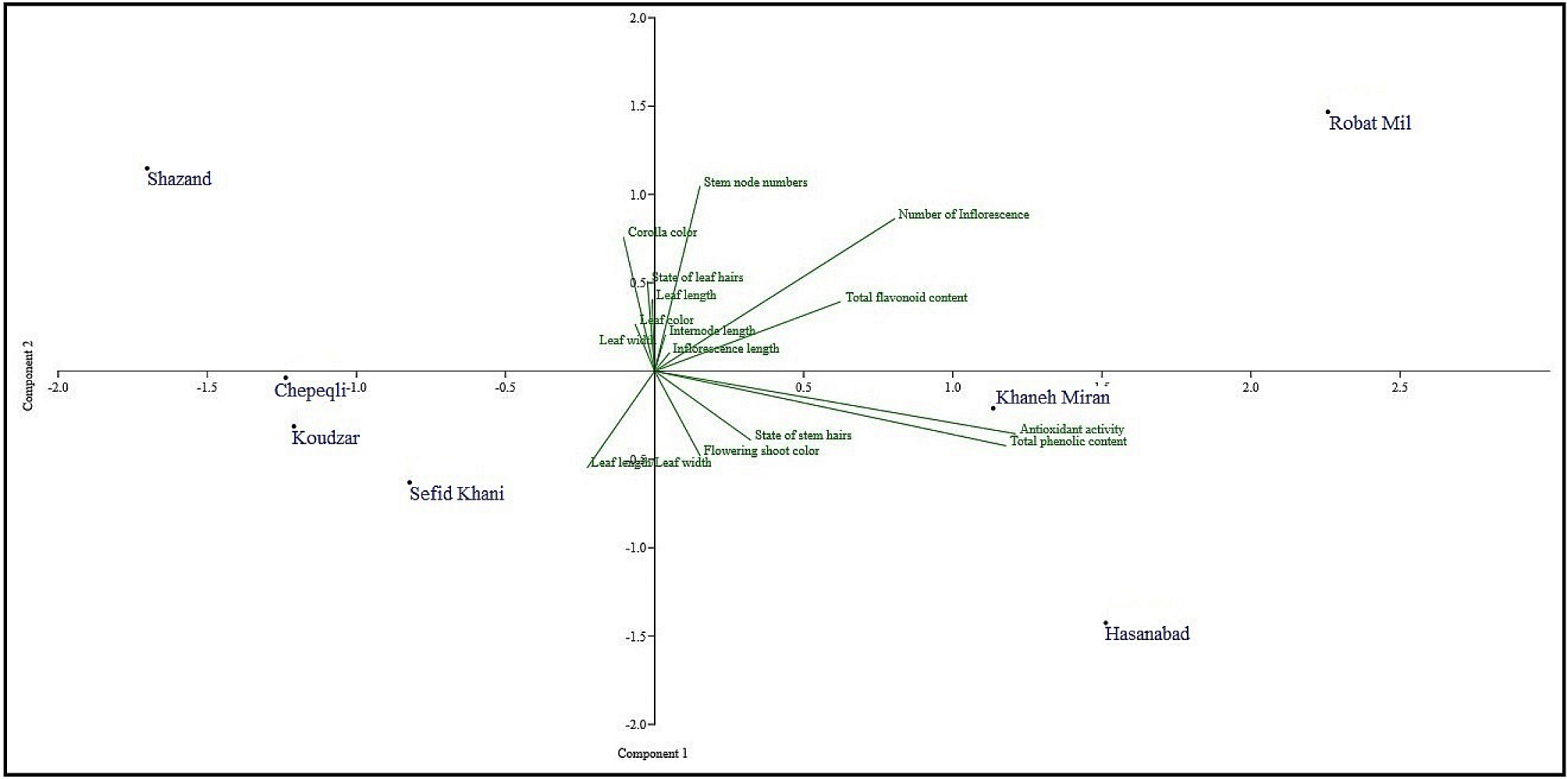
Biplot of PCA analysis in *Ajuga chamaecistus* populations based on morphological and biochemical traits

The population grouping in a biplot plot based on PC1 and PC2 may be related to their geographical distance. Thus, some populations of adjacent habitats were located in close areas on the biplot plot, which can be caused by similar environmental conditions and gene flow among these populations. It is worth noting that the accessions collected from Robat-Mil was correlated with the values of the measured characters, especially, stem node numbers, number of inflorescence, and total flavonoid content, whereas the accessions collected from Khaneh-Miran and Hasanabad correlated with total phenolic content, antioxidant activity and to a minor extent, state of stem hairs and flowering shoot color. Accessions collected from Koudzar, Chepeqli, and Sefid-Khani were very close, while other accessions were estranged (Fig. 7).

Currently, there is a lack of scientific information regarding the impact of climate change on *A. chamaecistus* in its natural habitats. However, previous research has indicated that global climate change plays a role in shaping the future distribution patterns of plant species [27]. There is increasing evidence suggesting that the average global temperature is rising, and precipitation levels are decreasing, primarily due to the increased emission of greenhouse gases [28]. Within our study habitats, there are *Thymus* species coexisting with *A. chamaecistus*. Previous studies have found that rising temperatures and reduced rainfall are the primary factors influencing the presence of *Thymus* species in these habitats [29]. Furthermore, it has been predicted that future climate change will lead to a significant reduction in suitable habitats for various species, including *Artemisia aucheri*, *Artemisia sieberi*, and *Daphne mucronata* in Iran [30, 31]. Therefore, the identification of suitable areas and desirable accessions can be an essential step for development of effective conservation programs.

Increasing human activities, such as agriculture, urbanization, and deforestation, contribute to the degradation and fragmentation of natural habitats. This habitat loss and fragmentation can have detrimental effects on the population size and genetic diversity of plant species, including *A. chamaecistus*. Reduced population sizes and genetic diversity make plants more vulnerable to environmental stressors and less capable of adapting to changing conditions [24]. Assessing the morphological and biochemical variation of *A. chamaecistus* can provide valuable insights into the current state of its populations and their potential resilience to habitat degradation. Different populations of *A. chamaecistus* may exhibit variations in growth habits, disease resistance, secondary metabolite production, and other important traits. By studying these variations, we can identify superior genotypes for cultivation and breeding purposes. This can lead to the development of improved varieties with enhanced medicinal properties, increased yields, and greater resilience to environmental stresses [32]. Assessing the morphological and biochemical variation of *A. chamaecistus* provides a foundation for targeted breeding efforts to meet the growing demand for this medicinal plant.

## Conclusions

In the present research, morphological and biochemical characteristics of *A. chamaecistus* were evaluated in different habitats of the Markazi province in the central part of Iran. Morphological analysis of *A. chamaecistus* showed high variation between qualitative and quantitative traits that help the breeder for selecting the desired genotype. The accessions collected from the Robat-Mil site showed the highest values of the examined morphological characteristics. Also, the populations of Robat-Mil, Hassanabad, and Khaneh-Miran were characterized by high values of total phenolic content, total flavonoid content, and antioxidant activity, which can be used in various industries, including pharmaceuticals, cosmetics, and food. Overall, the present results showed that the best area for the growth of *A. chamaecistus* with the production of significant contents of phenol and flavonoid is in Robat-Mil.

## Data Availability

The data that support the findings of this study are available from the corresponding author upon reasonable request.

## Declarations

### Statement specifying permissions

For this study, we acquired permission to study *A. chamaecistus* issued by the Agricultural and Natural Resources Ministry of Iran.

### Statement on experimental research and field studies on plants

The either cultivated or wild-growing plants sampled comply with relevant institutional, national, and international guidelines and domestic legislation of Iran.
